# Supplementary material for: Transcriptome Analysis Reveals Vimentin-Induced Disruption of Cell–Cell Associations Augments Breast Cancer Cell Migration
Source: Cells. 2022 Dec 13;11(24):4035. doi: 10.3390/cells11244035 (PMC9776984; doi:10.3390/cells11244035)
Supplement: Supplementary file 1 [file cells-11-04035-s001.zip › DEGs and enrichment analysis.zip!/DEGs and enrichment analysis/WT_VIMvsVector GO enrichment/GSEA/WT_VIM/ASTROCYTE_DIFFERENTIATION/ASTROCYTE_DIFFERENTIATION(GO_0048708).html]

Details for gene set ASTROCYTE\_DIFFERENTIATION(GO:0048708)[GSEA]


|  || Dataset | fpkm.sample |
| Phenotype | sample.cls |
| Upregulated in class | WT\_VIM |
| GeneSet | ASTROCYTE\_DIFFERENTIATION(GO:0048708) |
| Enrichment Score (ES) | 0.91412884 |
| Normalized Enrichment Score (NES) | 1.4725617 |
| Nominal p-value | 0.0 |
| FDR q-value | 0.16299984 |
| FWER p-Value | 0.163 |
Table: GSEA Results Summary

  

Fig 1: Enrichment plot: ASTROCYTE\_DIFFERENTIATION(GO:0048708)
  
Profile of the Running ES Score & Positions of GeneSet Members on the Rank Ordered List

  

| PROBE | DESCRIPTION   (from dataset) | GENE SYMBOL | GENE\_TITLE | RANK IN GENE LIST | RANK METRIC SCORE | RUNNING ES | CORE ENRICHMENT || 1 | ENSG00000125820 | NKX2-2 |  |  | 225 | Infinity | -0.0038 | Yes |
| 2 | ENSG00000087250 | MT3 |  |  | 1710 | Infinity | -0.0289 | Yes |
| 3 | ENSG00000026025 | VIM |  |  | 2388 | 1709.539 | 0.9141 | Yes |
| 4 | ENSG00000134323 | MYCN |  |  | 2737 | 6.503 | 0.9119 | No |
| 5 | ENSG00000105695 | MAG |  |  | 2953 | 5.112 | 0.9111 | No |
| 6 | ENSG00000180210 | F2 |  |  | 3166 | 4.318 | 0.9099 | No |
| 7 | ENSG00000050555 | LAMC3 |  |  | 3283 | 4.008 | 0.9102 | No |
| 8 | ENSG00000140538 | NTRK3 |  |  | 3371 | 3.836 | 0.9108 | No |
| 9 | ENSG00000186868 | MAPT |  |  | 3555 | 3.590 | 0.9098 | No |
| 10 | ENSG00000169071 | ROR2 |  |  | 3924 | 3.097 | 0.9053 | No |
| 11 | ENSG00000148400 | NOTCH1 |  |  | 4161 | 2.890 | 0.9029 | No |
| 12 | ENSG00000184486 | POU3F2 |  |  | 4174 | 2.884 | 0.9043 | No |
| 13 | ENSG00000146216 | TTBK1 |  |  | 4279 | 2.799 | 0.9041 | No |
| 14 | ENSG00000130164 | LDLR |  |  | 5405 | 2.231 | 0.8863 | No |
| 15 | ENSG00000110107 | PRPF19 |  |  | 6001 | 2.033 | 0.8773 | No |
| 16 | ENSG00000102882 | MAPK3 |  |  | 6737 | 1.867 | 0.8659 | No |
| 17 | ENSG00000123384 | LRP1 |  |  | 7159 | 1.782 | 0.8598 | No |
| 18 | ENSG00000172037 | LAMB2 |  |  | 7355 | 1.746 | 0.8575 | No |
| 19 | ENSG00000184144 | CNTN2 |  |  | 7444 | 1.726 | 0.8570 | No |
| 20 | ENSG00000115738 | ID2 |  |  | 7729 | 1.671 | 0.8531 | No |
| 21 | ENSG00000164690 | SHH |  |  | 8548 | 1.541 | 0.8401 | No |
| 22 | ENSG00000204305 | AGER |  |  | 8632 | 1.530 | 0.8395 | No |
| 23 | ENSG00000005513 | SOX8 |  |  | 8963 | 1.487 | 0.8348 | No |
| 24 | ENSG00000145191 | EIF2B5 |  |  | 9494 | 1.418 | 0.8266 | No |
| 25 | ENSG00000097007 | ABL1 |  |  | 10991 | 1.266 | 0.8020 | No |
| 26 | ENSG00000169032 | MAP2K1 |  |  | 11855 | 1.195 | 0.7880 | No |
| 27 | ENSG00000170075 | GPR37L1 |  |  | 12702 | 1.124 | 0.7743 | No |
| 28 | ENSG00000172201 | ID4 |  |  | 12983 | 1.103 | 0.7702 | No |
| 29 | ENSG00000131095 | GFAP |  |  | 14245 | 1.007 | 0.7494 | No |
| 30 | ENSG00000128271 | ADORA2A |  |  | 14382 | 0.996 | 0.7477 | No |
| 31 | ENSG00000100030 | MAPK1 |  |  | 15645 | 0.929 | 0.7268 | No |
| 32 | ENSG00000142192 | APP |  |  | 15899 | 0.926 | 0.7231 | No |
| 33 | ENSG00000184845 | DRD1 |  |  | 16190 | 0.907 | 0.7187 | No |
| 34 | ENSG00000116106 | EPHA4 |  |  | 16659 | 0.879 | 0.7112 | No |
| 35 | ENSG00000242689 | CNTF |  |  | 17037 | 0.855 | 0.7053 | No |
| 36 | ENSG00000183691 | NOG |  |  | 17189 | 0.846 | 0.7033 | No |
| 37 | ENSG00000196712 | NF1 |  |  | 17466 | 0.829 | 0.6990 | No |
| 38 | ENSG00000143546 | S100A8 |  |  | 18155 | 0.790 | 0.6878 | No |
| 39 | ENSG00000179295 | PTPN11 |  |  | 18193 | 0.787 | 0.6876 | No |
| 40 | ENSG00000007372 | PAX6 |  |  | 18507 | 0.771 | 0.6828 | No |
| 41 | ENSG00000133703 | KRAS |  |  | 19512 | 0.715 | 0.6662 | No |
| 42 | ENSG00000105810 | CDK6 |  |  | 21736 | 0.593 | 0.6289 | No |
| 43 | ENSG00000080815 | PSEN1 |  |  | 22258 | 0.559 | 0.6204 | No |
| 44 | ENSG00000175505 | CLCF1 |  |  | 22493 | 0.545 | 0.6167 | No |
| 45 | ENSG00000168610 | STAT3 |  |  | 22678 | 0.531 | 0.6139 | No |
| 46 | ENSG00000110693 | SOX6 |  |  | 22928 | 0.512 | 0.6100 | No |
| 47 | ENSG00000114315 | HES1 |  |  | 22995 | 0.506 | 0.6091 | No |
| 48 | ENSG00000134352 | IL6ST |  |  | 23242 | 0.492 | 0.6052 | No |
| 49 | ENSG00000163220 | S100A9 |  |  | 23819 | 0.453 | 0.5957 | No |
| 50 | ENSG00000136717 | BIN1 |  |  | 24085 | 0.430 | 0.5915 | No |
| 51 | ENSG00000146648 | EGFR |  |  | 24583 | 0.392 | 0.5833 | No |
| 52 | ENSG00000135919 | SERPINE2 |  |  | 25033 | 0.348 | 0.5759 | No |
| 53 | ENSG00000128342 | LIF |  |  | 25116 | 0.336 | 0.5747 | No |
| 54 | ENSG00000123560 | PLP1 |  |  | 25259 | 0.324 | 0.5725 | No |
| 55 | ENSG00000185483 | ROR1 |  |  | 25633 | 0.291 | 0.5663 | No |
| 56 | ENSG00000125398 | SOX9 |  |  | 25845 | 0.265 | 0.5629 | No |
| 57 | ENSG00000125845 | BMP2 |  |  | 26066 | 0.243 | 0.5593 | No |
| 58 | ENSG00000128602 | SMO |  |  | 26169 | 0.234 | 0.5577 | No |
| 59 | ENSG00000136869 | TLR4 |  |  | 26317 | 0.214 | 0.5553 | No |
| 60 | ENSG00000134198 | TSPAN2 |  |  | 26909 | 0.129 | 0.5454 | No |
| 61 | ENSG00000137270 | GCM1 |  |  | 29395 | 0.000 | 0.5033 | No |
| 62 | ENSG00000112333 | NR2E1 |  |  | 29667 | 0.000 | 0.4987 | No |
| 63 | ENSG00000171049 | FPR2 |  |  | 31331 | NaN | 0.4706 | No |
| 64 | ENSG00000162367 | TAL1 |  |  | 34918 | NaN | 0.4099 | No |
| 65 | ENSG00000173406 | DAB1 |  |  | 36812 | NaN | 0.3779 | No |
| 66 | ENSG00000197921 | HES5 |  |  | 55622 | NaN | 0.0594 | No |
| 67 | ENSG00000198719 | DLL1 |  |  | 56017 | NaN | 0.0528 | No |
| 68 | ENSG00000160307 | S100B |  |  | 57044 | NaN | 0.0354 | No |
Table: GSEA details
[plain text format]

  

Fig 2: ASTROCYTE\_DIFFERENTIATION(GO:0048708)
  
Blue-Pink O' Gram in the Space of the Analyzed GeneSet

  

Fig 3: ASTROCYTE\_DIFFERENTIATION(GO:0048708): Random ES distribution
  
Gene set null distribution of ES for
 **ASTROCYTE\_DIFFERENTIATION(GO:0048708)**
